# People Judge Discrimination Against Women More Harshly Than Discrimination Against Men – Does Statistical Fairness Discrimination Explain Why?

**DOI:** 10.3389/fpsyg.2021.675776

**Published:** 2021-09-20

**Authors:** Eberhard Feess, Jan Feld, Shakked Noy

**Affiliations:** ^1^School of Economics and Finance, Victoria Business School, Victoria University of Wellington, Wellington, New Zealand; ^2^Institute of Labor Economics, Bonn, Germany

**Keywords:** gender, discrimination, statistical fairness discrimination, employment discrimination (gender), moral judgments

## Abstract

Previous research has shown that people care less about men than about women who are left behind. We show that this finding extends to the domain of labor market discrimination: In identical scenarios, people judge discrimination against women more morally bad than discrimination against men. This result holds in a representative sample of the US population and in a larger but not representative sample of Amazon Mechanical Turk (Mturk) respondents. We test if this gender gap is driven by statistical fairness discrimination, a process in which people use the gender of the victim to draw inferences about other characteristics which matter for their fairness judgments. We test this explanation with a survey experiment in which we explicitly hold information about the victim of discrimination constant. Our results provide only mixed support for the statistical fairness discrimination explanation. In our representative sample, we see no meaningful or significant effect of the information treatments. By contrast, in our Mturk sample, we see that providing additional information partly reduces the effect of the victim’s gender on judgment of the discriminator. While people may engage in statistical fairness discrimination, this process is unlikely to be an exhaustive explanation for why discrimination against women is judged as worse.

## Introduction

Labor market outcomes of women are still worse than those of men. The gender wage gap is decreasing over time, but the ratio between full-time median salaries for women and men still varies between about 90% in Continental Europe and around 80% in the United States and the United Kingdom (Olivetti and Petrongolo, 2016; Ortiz-Ospina and Roser, 2018). Discrepancies in salaries are associated with differences in occupations, but are also found within the same occupation (see the overview by Kunze, 2018). In addition, field studies suggest that women are assigned to less challenging tasks than their male colleagues (De Pater et al., 2010; Bertrand, 2011; Chan and Anteby, 2016; Babcock et al., 2017), and are disadvantaged with regards to career opportunities (Allen et al., 2016) and dismissal decisions (Gupta et al., 2020).

Recent literature analyzes many potential channels for these differences, including differences in capital accumulation, preferences for professions, job descriptions, and competitiveness (see the overviews in Azmat and Petrongolo, 2014; Niederle, 2017). The literature also points to the role of stereotypes and discrimination (see the overview in Blau and Kahn, 2017). Laboratory experiments explore discrimination in controlled environments, and find that women with the same performance are less likely to be hired for male-stereotyped tasks (Reuben et al., 2014; Bohnet et al., 2016; Coffman et al., 2018). According to the European Working Conditions Survey, 3.2% of women report having been subjected to discrimination at work on the basis of their sex in the preceding 12 months, compared to only 1.1% of men (Eurofund, 2018).

Notably, however, women have also overtaken men in some domains. Across most OECD countries, women are now more likely to graduate from university (OECD, 2020). There are growing concerns about the job prospects of low-skilled men, who have seen a significant reduction in real income in the US, and many of whom have left the labor force (Autor and Wasserman, 2013; Binder and Bound, 2019). There is also a growing literature documenting a gender bias against men in several fields (Booth and Leigh, 2010; Breda and Hillion, 2016; Bohren et al., 2019).

Against this backdrop, we investigate how the gender of the victim of discrimination affects people’s moral judgment about the discriminator. We answer this question using a survey experiment with two samples. Our main sample consists of 478 respondents who are representative of the US population in terms of gender, age, race, education and political orientation. Our replication sample consists of 1,169 US based respondents recruited from Amazon Mechanical Turk (Mturk).^1^ For this study, we define gender discrimination as hiring someone of one gender *despite knowing that an applicant of the opposite gender is more qualified and more productive*. In our base treatment, each respondent is shown two scenarios (in random order) in which a manager discriminates, and is asked to evaluate managers’ decisions on a scale that ranges from 0 “very morally wrong” to 100 “very morally right.” In one of these scenarios, the manager discriminates against a woman, and in the other against a man.

We use these judgments to estimate the effects of the victim’s gender on the moral evaluation of the actions of the discriminator in two ways. The first is the (within-subject) *pro-women attitude*, which we define as the difference in judgment of a manager who discriminates against a woman compared to the judgment of a manager who discriminates against a man *for a particular respondent*. Using this measure, we find that respondents judge discrimination against women on average 5.5 points more morally wrong. This measure is based on respondents’ answers to two scenarios - presented right after each other – in which only the gender of the victim differs. By judging these two related scenarios, however, respondents may feel the need to be consistent, which would reduce the measured pro-women attitudes. For example, having just judged discrimination against a woman as very morally bad may compel respondents to also judge discrimination against a man as very bad. By contrast, in many real world applications respondents only judge one case of discrimination at a time. We therefore also report a *between-subject pro-women attitude*, which is the difference in judgment of discrimination against a woman compared to discrimination against a man, based on respondents’ judgment of the scenario they saw first. The between-subject pro-women attitude is 11.8 points, which is substantially larger. We replicate both of these results with our Mturk sample.

We further investigate potential reasons for the pro-women attitude. This investigation is inspired by a recent study by Cappelen et al. (2019), who ask whether people are less concerned about men falling behind than about women falling behind. In their experiment, observers can redistribute money from women who win to losing men and vice versa. Observers are less likely to redistribute money to low-performing men, suggesting that they are indeed more accepting of men falling behind. Interestingly, this gender gap disappears when losers and winners are determined by chance instead of their performance in a real effort task. The authors interpret this result as evidence for statistical fairness discrimination, that is, that people use gender as a signal for unobserved characteristics which matter for their fairness judgments. In the experiment, people who engage in statistical fairness discrimination may be less likely to help men because they believe that men who have fallen behind have worked less hard. This interpretation is consistent with earlier findings by Cappelen et al. (2007) that many people believe productivity differences justify wage differentials if and only if they reflect different effort.

We explore the role of statistical fairness discrimination in explaining the average pro-women attitude with an embedded survey experiment. We ask randomly selected respondents to judge additional scenarios that are either very similar to the base scenarios (control group) or explicitly state that the job is in an industry without gender discrimination, that the man and woman who applied for the job worked equally hard in their career, and that both applicants would suffer equally from not getting the job (treatment group).

The results of this survey experiment show only mixed support for the statistical fairness discrimination hypothesis. In our main sample with Qualtrics respondents, we see no evidence that the additional information changes respondents’ pro-women attitude. The average pro-women attitudes in the treatment and control groups are very similar, and none of the differences are statistically significant. By contrast, in our replication sample with Mturk respondents, we do see that providing additional information significantly reduces the pro-women attitude of respondents who exhibited a positive pro-women attitude in the base scenarios. However, even in scenarios in which we hold applicants’ effort, suffering, and exposure to discrimination constant, pro-women respondents still show a statistically significant pro-women attitude. While statistical fairness discrimination may play a role in explaining differences in judgments about discriminated women and men, it is unlikely to be the whole story.

The concept of statistical fairness discrimination as defined by Cappelen et al. (2019) builds on the more general and widely discussed concept of statistical discrimination (Phelps, 1972; Arrow, 1973). In both concepts, observable characteristics of people are used to infer unobservable, but relevant, characteristics. Traditional statistical discrimination may enhance efficiency, but it also violates widespread fairness norms. A famous and highly controversial example is the finding by Knowles et al. (2001) that police checking for illegal drugs are more likely to search cars of Black than White drivers, which the authors argue equilibrates the detection probabilities for the two groups at the margin. Many forms of statistical discrimination are prohibited. In many countries it is illegal to use race, sex, age or disability as criteria for decisions on hiring or promotion, even if these characteristics predict performance. While traditional statistical discrimination involves a tension between efficiency and fairness, statistical fairness discrimination is based on fairness considerations itself. It builds on the idea people use group characteristics to draw inferences about unobserved characteristics (e.g., deservingness of help) that matter for their fairness judgments.

The concept of statistical fairness discrimination may be useful for a better understanding of a wide range of differences in people’s social preferences (see the overview by Eckel and Grossman, 2008). Many papers find that subjects care more about women in social dilemma situations (FeldmanHall et al., 2016), that defendants killing women are far more likely to be sentenced to death than defendants killing men (Shatz and Shatz, 2012), and that subjects give more to women in dictator games (Engel, 2011). While these observations may just reflect people caring more about the wellbeing of women (see the literature review in Eagly and Mladinic, 1994), they might also reflect statistical fairness discrimination in the sense that, for example, women are seen as more vulnerable.

Our finding that people are less concerned about discrimination against men than women relates to a paper by Block et al. (2019). Their paper first shows that people are more concerned about the underrepresentation of women in male-dominated careers (Science, Technology, Engineering, and Math) than about men in female-dominated careers (Healthcare, Early Education, and Domestic roles). They derive three main insights on the reasons for this difference: First, people believe that underrepresentation only deserves countervailing measures if it is based on discrimination rather than on preferences. As men are perceived as being not interested in female-dominated careers, there seems to be no reason to worry about their underrepresentation. Second, female-dominated careers are viewed as less prestigious, so that underrepresentation of males is not interpreted as a disadvantage. Third, differences in salaries hardly matter for peoples’ different concerns.

In another set of studies, Winegard et al. (2018) document that liberals’ judgments favor groups they perceive to be disadvantaged, like women and Black people. Their approach is similar to ours in that they compare judgments of identical situations in which one key demographic characteristics differs (see also Stewart-Williams et al., 2021). For example, they show liberals trust otherwise identical scientific studies more if the results are favorable for disadvantaged groups (women and Blacks) than privileged groups (men and Whites). These differences in judgments are consistently predicted by Equalitarianism – the belief that differences between demographic groups are not driven by biological factors but by prejudice, and that society can and should make all groups equal.

In another related paper, Haaland and Roth (2021) use a representative sample of the US household population to analyze beliefs about racial discrimination, and investigate how these beliefs are correlated with the view on affirmative action. They also find that providing different kinds of information influences people’s perceptions of discrimination. Interestingly, the authors show that, while providing accurate information changes the beliefs on the actual degree of discrimination, it has only little impact on the view on affirmative action.

Overall, our paper makes three main contributions. First, we add to a body of research showing that many people show more concern for disadvantaged groups than advantaged groups. While these studies are typically done with convenient samples (e.g., from Mturk) we show that this conclusion also holds in a representative sample. Second, we carefully investigate to what extent these differences in judgment are driven by statistical fairness discrimination – an explanation which has only received limited attention in the literature. Our embedded survey experiment lends some, but not very strong support for this explanation. Third, our comparison of the within-subject and between-subject results reveals that people’s judgments are influenced by the tension between finding discrimination against women worse and the normative view that the gender of the victim should not affect their judgments.

## Sample

Our main analysis is based on a sample of 478 respondents recruited by Qualtrics. Respondents participated between 4 June 2020 and 30 June 2020. This main sample is representative of the population of US adults in terms of sex, age, education, and political orientation. Qualtrics achieved this representativeness by recruiting respondents whose characteristics match population statistics taken from the 2018 American Community Survey (for sex, age in bins^2^, education, and race, see United States Census Bureau, 2021) and a May 1–13, 2020 Gallup survey (for political orientation, see Gallup, 2021). Representativeness targets were reached for all of these characteristics, except that the mean age in our sample is 3 years under the mean age of over-18 Americans, mainly due to undersampling of people in the over-65 age bin.

Table 1 shows summary statistics for our main estimation sample (based only on Qualtrics respondents). Respondents are on average 46 years old; 51% are female, 74% are White and 12% are Black; 38% have a high school degree or less and 12% have a graduate degree. The political leaning of the respondents is measured with the following Gallup question: “Generally speaking, do you think of yourself as a Republican, Democrat, Independent, or what?”. 31% of respondents identify as Democrats, 28% as Republican, and 36% as Independents. The data and Stata do-files to create Table 1 and all other results shown in our paper are available following this link: osf.io/2eq43.

**TABLE 1:** Summary statistics (*N* = 478).

	**(1)**	**(2)**	**(3)**	**(4)**	**(5)**
	
	**Mean**	***SD***	**Min**	**Max**	**Target mean**
Age	46.18	17.37	18	100	49.68
Female	0.51	0.50	0	1	0.51
*Race*					
White	0.74	0.44	0	1	0.74
Black	0.12	0.33	0	1	0.12
Asian or Pacific Islander	0.06	0.23	0	1	0.06
Amer. Indian or Alaska Native	0.01	0.10	0	1	0.01
Other	0.07	0.25	0	1	0.07
*Educational attainment*					
High school or less	0.38	0.49	0	1	0.40
Some college	0.23	0.42	0	1	0.23
Associates degree	0.08	0.28	0	1	0.08
Bachelor’s degree	0.18	0.39	0	1	0.18
Graduate degree	0.12	0.32	0	1	0.11
*Political orientation*					
Democrat	0.31	0.46	0	1	0.31
Republican	0.28	0.45	0	1	0.28
Independent	0.36	0.48	0	1	0.37
Other	0.04	0.20	0	1	0.04

*These summary statistics are based on our Qualtrics sample. Column (5) shows the target means for each variable which are based on a Gallup survey for political orientations and the 2018 American Community Survey for all other variables.*

Our Qualtrics sample is not representative of the US adult population along all dimensions. For example, some people in the US could not have made it into our sample because they do not speak English, or they do not have access to the internet. We therefore do not interpret our results as unbiased estimates of the relevant population parameters. However, having representativeness along several key dimensions gives us confidence that the direction of our point estimates will also hold in the general population.

To be able to test the robustness of our results, we additionally collected data from respondents recruited on Mturk. These respondents filled in a shorter version of the questionnaire, which included the same scenarios as in the Qualtrics survey but excluded some questions about beliefs and demographics. By shortening the questionnaire, we could stretch our research budget and increase the total number of Mturk respondents in our estimation sample to 1,169. This estimation sample excludes 13 respondents who indicated that they were less than 18 years old. Mturk respondents filled in a shorter version of the survey between 30 July 2020 and 22 August 2020. This sample is not representative of the US population. Respondents in our Mturk sample are on average 37 years old, 50% female and more educated than our main sample (see Supplementary Appendix Table 1 for more summary statistics).

## How Does the Gender of the Victim Affect People’s Moral Judgment About the Discriminator?

### The Survey

**FIGURE 1:**
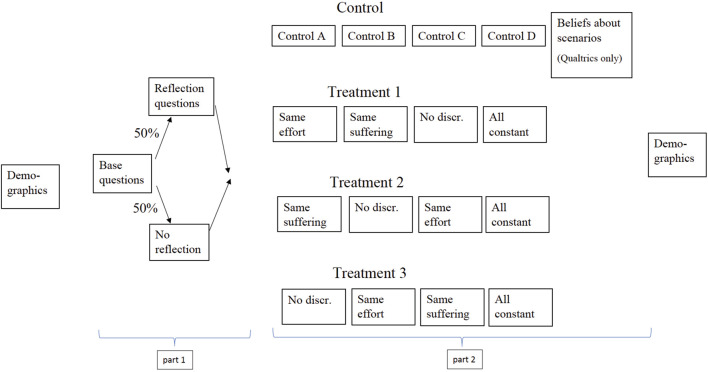
Structure of survey.

Figure 1 shows the structure of the survey. In this section, we will describe the questions relating to the first part of our analysis. We will describe the questions relating to the second part of our analysis in Section “Predictions.” The complete survey text is available in Supplementary Appendix B.

#### Judging Discrimination in Two Base Scenarios

**FIGURE 2:**
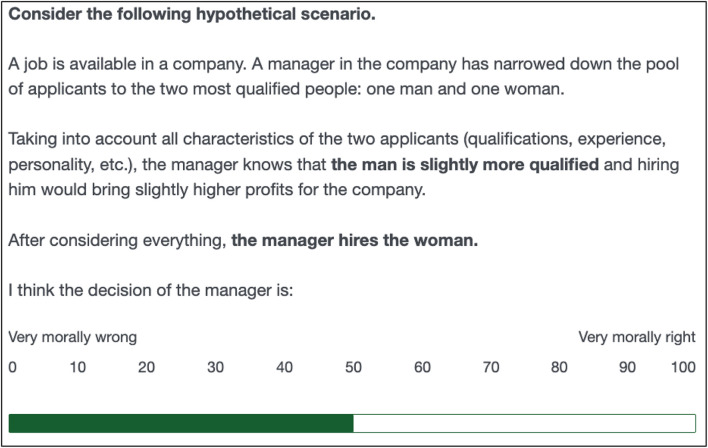
Screenshot of discrimination-against-women base scenario.

In the first part of the survey, both Qualtrics and Mturk respondents were asked to judge discrimination in two scenarios (“Base questions”). These scenarios consist of situations in which a manager has to decide between giving the job to a man or a woman. In one scenario, the manager discriminates against the woman and in the other scenario the manager discriminates against the man. More specifically, the discrimination-against-the-woman scenario states that “*[t]aking into account all characteristics of the two applicants (qualifications, experience, personality, etc.), the manager knows that the* ***woman is slightly more qualified*** *and hiring her would bring slightly higher profits for the company. After considering everything*, ***the manager hires the man.”*** (see screenshot of the whole scenario text in Figure 2). The discrimination-against-the-man scenario was identical except for the man being slightly more qualified and the manager hiring the woman. The order of those two scenarios was randomly assigned. For each scenario, respondents were asked to judge the manager’s decision on a scale that ranges from 0 “very morally wrong” to 100 “very morally right”.

For ease of interpretation, we center and reverse the judgments scores shown in this figure so that they range from –50 (very morally right) over 0 (neutral) to +50 (very morally wrong).

#### Follow-Up Questions to Clarify Judgments

After judging discrimination in the two base scenarios, a randomly selected 50% of respondents were asked to confirm their judgments from the first two scenarios. More specifically, the questionnaire showed a different follow-up question for each of the following three types of respondents: (1) Those who judged discrimination against women more negatively, (2) those who judged discrimination against women and men equally bad, and (3) those who judged discrimination against men more negatively. Each of these types of respondents was given the possibility to confirm their initial judgments. For example, respondents who judged discrimination against women more negatively saw the following text: *“Your evaluations of the manager’s decisions in these two scenarios suggest that: You find it worse (from a moral perspective) if a manager hires a less qualified man over a more qualified woman (compared to the other way around).”* Respondents could then clarify their evaluations of the first two scenarios by choosing one of the following three answer options: “*Yes, this is correct*,” “*No, I find both equally bad (or good)*,” or “*No, I find it worse if the manager hires a less qualified woman over a more qualified man.*”

### Measures of the Effect of the Victim’s Gender on Judgments About Discrimination

We use two methods to measure the effect of the victim’s gender on the judgment of the discriminator. Our first measure consists of the judgment of a manager who discriminates against a man minus the judgment of a manager who discriminates against a woman *within each respondent*. We will refer to this difference as within-subject pro-women attitude, or with the shorthand “pro-women attitude.” For example, a respondent who judges discrimination against a woman with a score of 10 (somewhat morally wrong) and discrimination against a man with a score of 0 (neutral) has a pro-women attitude of 10 points. Negative values of this measure show pro-men attitudes. Besides computing the average pro-women attitude, we will also show their distribution. Based on the pro-women attitude in the base scenario, we classify respondents as “pro-women” if their pro-women attitude is larger than 1 point, as “neutral” if their pro-women attitude is between –1 and +1 points, and as “pro-men” if their pro-women attitude is below –1 point.

Our second measure of the effect of the victim’s gender relies on the judgment of the first scenario respondents saw (where the gender of the victim was randomized across subjects), which allows us to calculate the *between-subject pro-women attitude.* This measure is equal to the average judgment of managers who discriminate against a man minus the average judgment of managers who discriminate against a woman, *across different subjects*. Naturally, we cannot calculate this measure for individual respondents.

The difference between these two measures allows us to infer to what extent respondents themselves believe that the gender of the victim should not affect their judgment. The key to identifying this belief is that respondents can adjust their second judgment to be consistent with their first judgment. For example, assume that respondents find on average discrimination against women intuitively worse, but also believe that the victim of the gender should not affect their judgments. In this case the between-subject pro-women attitude will reveal the intuition of the respondents: in the first scenarios they see, respondents would judge a discriminator more harshly if the victim is a woman. However, if respondents also hold the normative view that the gender of the victim should not matter, they would adjust their second judgment to be consistent with their first judgment. If this adjustment is complete, we would observe no pro-women attitude with our within-subject measure. However, such adjustment would be revealed by order effects. Respondents who see scenarios describing discrimination against a woman first should judge the manager in *both* scenarios more harshly: in the first scenario because they feel discrimination against woman is particularly bad, and in the second because they feel compelled to judge discrimination similarly harshly if the victim is a man. Using such differences between within and between subject judgments is a tool commonly used in psychological studies to draw inferences about conflicting motivations (e.g. Uhlmann et al., 2009; Winegard et al., 2018).

### Results

#### Between-Subject and Within-Subject Pro-women Attitudes

When considering all judgments in the base scenarios, we see that respondents judge discrimination against a woman as 5.5 points more morally bad than discrimination against a man (10.5 points vs. 5.0 points). A one-sample *t*-test confirms that the average of this within sample pro-women attitude differs significantly from zero (*p*-value: < 0.001).

Table 2 shows the average judgment of the manager in scenarios in which a woman was discriminated and in which a man was discriminated, separately for respondents who randomly saw a discriminated woman (Columns 1 and 2) and man first (Columns 3 and 4). Focusing on the first judgments (Columns 1 and 3), we see a substantial between-subject pro-woman attitude. Respondents evaluate discrimination against a woman 11.8 points more morally wrong than discrimination against a man (13.3 points vs. 1.5 points). A two-sample *t*-test shows that these judgments are significantly different from each other (*p*-value < 0.001). When judging discrimination in isolation, respondents judge managers who discriminate against a woman substantially more harshly.

**TABLE 2:** Estimating the between-subject pro-women attitude and order effects (Qualtrics sample).

	**(1)**	**(2)**	**(3)**	**(4)**
	
	**Woman discr. (1st judgment)**	**Man discr. (2nd judgment)**	**Man discr. (1st judgment)**	**Woman discr. (2nd judgment)**
Av. judgment in each	13.3	9.0	1.5	8.0
Av. judgment in both (Col 1, 2 and Col 3, 4)	11.1		4.7	
Order effect	6.4			
Between-subject pro-women attitude	11.8			

*All values refer to the reversed and centered judgment score. Higher values indicate finding discrimination more morally bad.*

Table 2 also reveals that there are order effects. Despite seeing two identical scenarios, respondents judge the behavior of the manager in both scenarios on average 6.4 points more morally wrong if they first saw the scenario with the discriminated woman (11.1 points vs. 4.7 points). Following Uhlmann et al. (2009) and Winegard et al. (2018), we interpret this order effect as evidence that on average respondents themselves think that the gender of the victim should matter less than the between-subject pro-women attitude reveals. The within-subject pro-woman attitude therefore only shows the part of the pro-woman attitude which respondents are comfortable revealing (either to themselves or the researcher).

**FIGURE 3:**
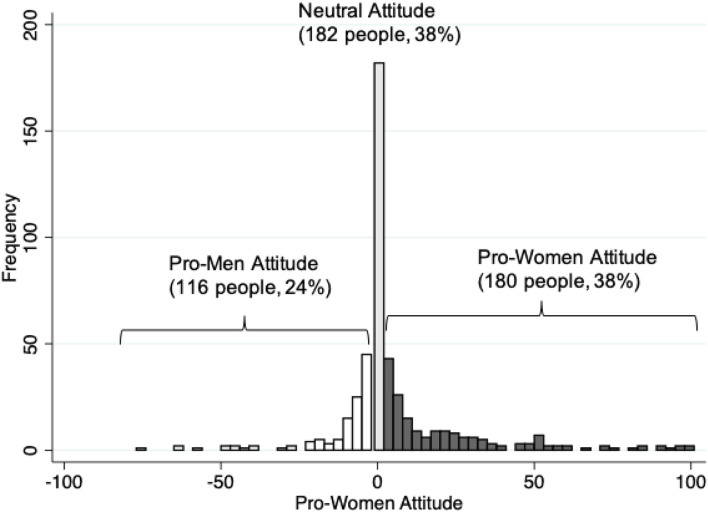
Distribution of pro-women attitude. This figure shows the pro-women attitude of 478 respondents in the Qualtrics sample. The pro-women attitude of each respondent is the moral judgment about a manager who discriminates against a woman (on a –50 to +50 scale where higher values indicate more disapproval) minus the moral judgment about a manager who discriminates against a man. Positive values mean respondents judge discrimination against a woman as more morally bad.

Figure 3 shows the distribution of the within-subject pro-women attitude. Based on this measure, we classify 38% of respondents as pro-women, 38% of respondents as neutral, and 24% of respondents as pro-men. Furthermore, pro-women respondents feel more strongly than pro-men respondents. On average pro-women respondents judge discrimination against women 22.4 points more morally bad, while pro-men respondents judge discrimination against men only 12.3 points more morally bad.

The distribution of the pro-women attitude shown in Figure 2 partly reflects measurement error because not all respondents can precisely state their views using sliders in an online questionnaire. If such measurement error is random — and we believe that is most plausible — it should not affect the average pro-women attitude in our sample. However, it would increase the variance of our measure of the pro-women attitude. Random measurement error would also cause us to wrongly classify some respondents’ views. Take, for example, a respondent who finds discrimination against women and men equally bad. Having just judged a scenario in which a woman is discriminated, this respondent may not remember the exact position of the slider on the previous page and by chance judge discrimination against a man as worse. We would wrongly judge such a respondent as being pro-men.

Measurement error is not a concern when considering respondents’ self-classifications. Based on answers to the follow-up question, the share of self-classified neutral respondents increases to 43%; leaving us with 34% self-classified pro-women and 22% self-classified pro-men respondents. Besides measurement error, the higher share of neutral respondents may also be triggered by the chance to reflect on their previous judgments.

**FIGURE 4:**
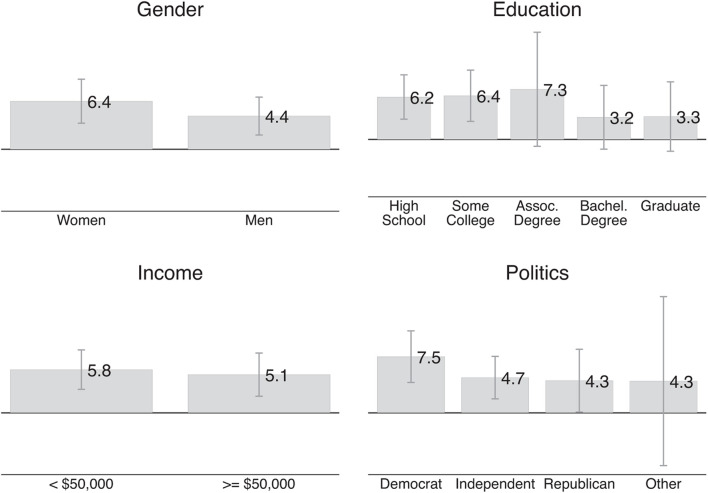
Demographic predictors of pro-women attitude. This figure shows the average pro-women attitude for different groups in our Qualtrics sample. The pro-women attitude of each respondent is the moral judgment about a manager who discriminates against a woman (on a –50 to +50 scale where higher values indicate more disapproval) minus the moral judgment about a manager who discriminates against a man. Positive values mean that respondents judge discrimination against a woman as more morally bad.

Figure 4 shows different average values of pro-women attitude along the lines of gender, education, income, and political orientation. Women’s average pro-women attitude is 6.4 points and men’s average pro-women attitude is 4.4 points; the average pro-women attitude by level of education ranges from 3.2 points for respondents with a Bachelor’s degree to 7.3 points for respondents with an Associate’s degree; respondents earning below $50,000 have a very similar pro-women attitude to respondents who earn $50,000 + per year (5.8 points vs. 5.1 points). As expected, Democrats have 7.5 points stronger pro-women attitude than Republicans, but even the Republicans’ pro-women attitude is positive (4.3 points). However, *F*-tests reveal that none of the aforementioned differences is statistically significant.

#### Replication With Mturk Sample

While the magnitudes differ, all our key results replicate with our non-representative Mturk sample. In this sample, respondents show on average a statistically significant pro-women attitude of 4.1 points (one sample *t*-test, *p*-value < 0.001). We classify 43% of respondents as pro-women, 30% as neutral, and 27% as pro-men. When giving respondents the chance to clarify their view, the percentage of neutral respondents increases to 37%; leaving 36% pro-women, and 27% pro-men respondents. The between-subject pro-women attitude is a statistically significant 6.2 points (two sample *t*-test, *p*-value < 0.001), which is substantially larger than the within-subject pro-women attitude of 4.1 points.

## Does Statistical Fairness Discrimination Drive Respondents’ Pro-Women Attitude?

In our base scenarios, we stated that the manager hires a woman instead of a more productive man or vice versa. We neither gave reasons for the productivity difference nor mentioned explicitly that the two applicants are otherwise in identical situations. A plausible explanation for the pro-women attitude is hence that respondents have engaged in statistical fairness discrimination: Respondents may use the gender of the victim of discrimination as a signal for other unobserved characteristics of the situation which affect their judgment of the discriminator.

### The Survey Experiment and Questions About Beliefs

We investigate the role of beliefs about unobserved characteristics using an embedded survey experiment. After judging the base scenarios, each respondent saw four additional pairs of scenarios that were again identical except for the victim’s gender. Each pair of scenarios was shown on the same page allowing respondents to easily compare their judgments about managers who discriminate against a woman and managers who discriminate against a man.

Half of respondents were randomly assigned to the control group. These respondents saw scenarios that only differed from the base scenarios in the location of the job (urban area, suburban area, rural area, major city). We added this arguably irrelevant piece of information to avoid showing scenarios identical to the base scenario. The other half of respondents were randomly assigned to one of three treatment arms. Respondents in each treatment arm saw scenarios describing jobs in the same locations as the control group. Besides seeing the same locations, respondents in each treatment arm saw the same additional texts. The “same effort” text stated that the woman and man under consideration worked equally hard to get the job; the “same suffering” text stated that they would suffer equally from not getting the job; the “no discrimination” text stated that the job is in an industry without gender discrimination, and the “all constant” treatment combines the previous three texts. Table 3 shows the exact wording of all treatment texts.

**TABLE 3:** Treatment text for survey experiment.

**Treatment name**	**Treatment text**
Same effort	**The man and the woman have worked equally hard in their career.** For example, both regularly studied on the weekends while their friends were out partying.
Same suffering	**The man and the woman would suffer equally much from not getting the job.** For example, both are currently unemployed, but have enough savings so that they could go without getting a paycheck for another 4 weeks. Also, both would find it equally hard to get a new job. Neither of them has to support a family.
No discrimination	**The job is in an industry where there is no gender discrimination.** A number of studies have convincingly shown that in this industry neither men nor women face discrimination in hiring decisions, nor do they face any other unfair treatment by coworkers or supervisors because of their gender.
All constant	**The man and the woman would suffer equally much from not getting the job, the man and the woman have worked equally hard in their career, and the job is in an industry with no gender discrimination.**

*We use **bold** here as we did in the actual survey.*

**FIGURE 5:**
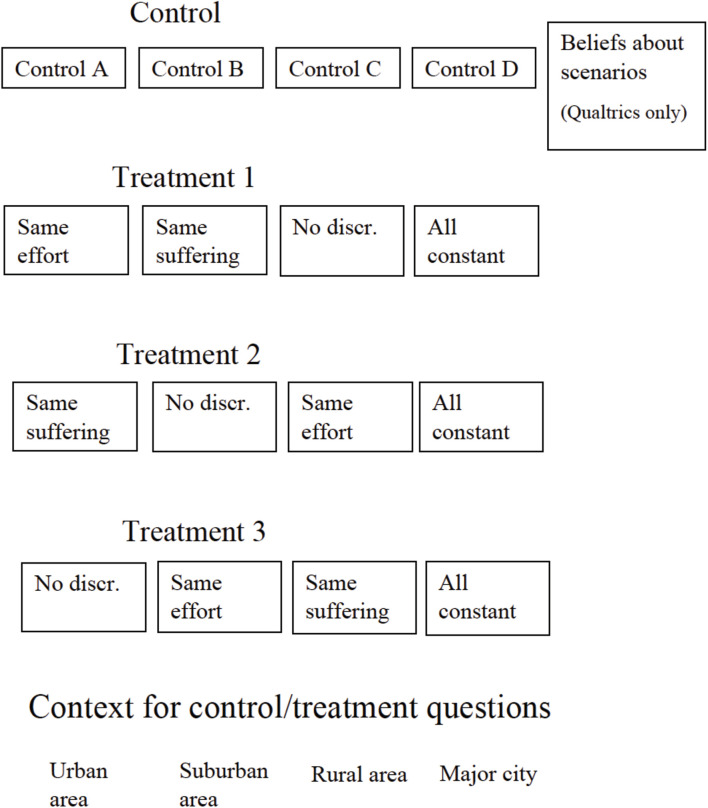
Structure of embedded survey experiment.

Figure 5 shows the structure of the experiment. For each of the treatment arms, the first three information treatments consisted of the “same effort,” “same suffering,” and “no discrimination” texts. The order of these texts differed between treatment arms to prevent order effects from driving our results. Each of these three information treatments appears in the first set of scenarios in one treatment arm, in the second set of scenarios in another treatment arm, and in the third set of scenarios in another treatment arm. All three treatment arms saw the “all constant” treatment last. We conducted the survey experiment with the Qualtrics and Mturk samples.

Respondents in the control group first completed the survey experiment and were then asked for their beliefs about the women and men in the previous scenarios. In particular, we asked to which extent the women or men in the previous scenarios (1) would have suffered more from not getting the job, (2) worked harder to get where they are in their career, (3) are generally more hard-working (in their career and other aspects of their life), and (4) would be more discriminated against in the labor market. We elicited these beliefs only in the Qualtrics sample.

### Predictions

If statistical fairness discrimination drives respondents’ within-subject pro-women attitude, we should see two patterns in the data. First, we should see that the information texts should lead to more gender-neutral judgments about discrimination compared to the control group. Thus, holding suffering and effort of both candidates constant as well as stating that the job is in an industry without gender discrimination should reduce the pro-women attitude of pro-women respondents and increase the pro-women attitude of pro-men respondents (i.e., reduce their *pro-men* attitude). The pro-women attitude of neutral respondents should not be affected.

Second, respondents that we classified based on their answers in the first part of the survey as pro-women and pro-men should believe that the women and men described in the scenarios differ along characteristics that make discrimination against their favored gender more objectionable. In particular, we would expect that pro-women respondents believe that the women described in the control scenarios would have worked harder in their career and in general, would suffer more from not getting the job, and would have suffered more discrimination. Pro-men respondents should hold beliefs in opposite directions.

### Analysis of Survey Experiment

We analyze the results of the survey experiment by comparing the mean within-subject pro-women attitude between respondents in the control and treatment scenarios *separately for* pro-women, neutral, and pro-men respondents. More specifically, we estimate three separate regressions, one for each group of respondents. In each regression, the dependent variable is the within-subject pro-women attitude in the survey experiment. Independent variables are four treatment indicators (one for each information treatment), leaving the control group as our comparison group. The coefficients of the treatment indicators show the mean differences between the pro-women attitude in a given treatment compared to the control group. For example, the “same effort” coefficient shows the difference between the mean pro-women attitude in the control group (across all four scenarios) and the mean pro-women attitude in the scenarios which contained the “same effort” text (the first scenario in Treatment 1, the third scenario in Treatment 2, and the second scenario in Treatment 3). For all three regressions, we cluster our standard errors at the individual level. This way of clustering accounts for the fact that we observe multiple pro-women attitudes for each respondent.

We report our results by showing the mean pro-women attitude in the control group and each treatment group, again separately for all three groups of respondents. These means directly relate to our regression coefficients. For the control group, the mean pro-women attitude is equal to the constant. For the treatment groups, the means are equal to the constant plus the respective treatment coefficient.

### Results

#### Effects of Information Treatments on Pro-women Attitude

**FIGURE 6:**
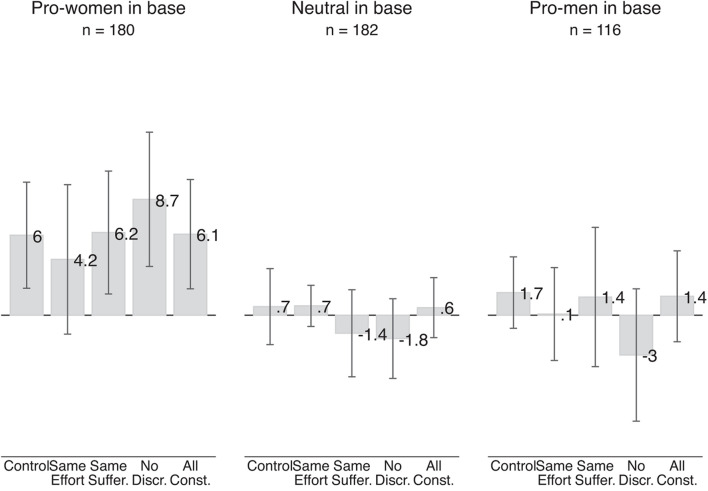
Effect of information treatments on pro-women attitude (Qualtrics sample). The gray bars show the average pro-women attitude in a given treatment. The left-most bar is based on 688 judgments made by 86 respondents who were pro-women in the base scenarios and were randomly assigned to the control group, judging four pairs of scenarios involving two judgments each. Each of the next four bars is based on 188 judgments made by 94 respondents who were pro-women in the base scenarios and were randomly assigned to any of the three treatment groups. Similarly, the left-most bar in the middle section is based on 744 judgments by 93 respondents who were neutral in the base scenario, and each of the next four bars is based on 178 judgments made by 89 respondents. The left-most bar in the right-most section is based on 480 judgments made by 60 respondents who were pro-men in the base scenario, and each of the next four bars is based on 112 judgments by 56 respondents. Vertical lines show 95 percent confidence intervals based on standard errors clustered at the individual level.

Figure 6 shows the results of the survey experiment for the Qualtrics sample. The gray bars show the average between-subject pro-women attitude in the control group and for the various information treatments; separately for respondents who we classified, based on their responses to the base scenarios, as pro-women, neutral, or pro-men.

Overall, we see no meaningful or statistically significant effect for any of our three groups of respondents. Pro-women respondents show an average pro-women attitude of 6 points in the control scenarios and an almost identical pro-women attitude of 6.1 points in scenarios which held effort and suffering of the male and female applicant constant as well as describing a job in an industry without gender discrimination (All constant treatment). While point estimates differ slightly, we also see no evidence of an effect in any of the other information treatments: holding suffering, effort or discrimination individually constant has no meaningful effect on pro-women attitude. Three of the four point estimates (same suffering, no discrimination, all constant) even suggest that the information treatments increased respondents’ pro-women attitude. These results go against our predictions.

For neutral respondents, we also see no impact of any of the information treatments. They show an average pro-women attitude of 0.7 points in the control scenarios, which is almost identical to the pro-women attitude of 0.6 points in the scenarios which held effort, suffering and discrimination constant. None of the average pro-women attitudes are significantly different from zero. These results are in line with our predictions. Holding reasons for finding discrimination against one gender more objectionable constant does not affect the pro-women attitude of respondents who already judged discrimination against women and men as equally bad.

For pro-men respondents, we also see no significant changes in their pro-women attitude in response to any of the information treatments. This result is driven by the control group. Respondents with an initial pro-men attitude in the base scenarios who were randomly assigned to the control group now find discrimination against women slightly more bad: They show a positive pro-women attitude of 1.7 points. Neither this pro-women attitude nor any of the pro-women attitudes in the treatment scenarios are significantly different from zero. These results are again inconsistent with our predictions.

What could be driving the increase of the pro-women attitude (i.e., reduction of pro-men attitude) of respondents who we initially classified as pro-men? Part of this increase is likely be driven by regression to the mean. Some respondents classified as pro-men may in fact be neutral or pro-women but have, by chance, moved the slider to indicate that they find discrimination against men more morally wrong. When evaluating similar subsequent scenarios, those respondents may have on average reverted back to their true value of pro-women attitude.^3^ It may also be that these respondents feel increasingly uncomfortable of revealing their pro-men attitudes to us as the researchers. Whatever the reasons for this reduction are, pro-men respondents’ pro-men attitude from the base scenario is not stable. In subsequent scenarios in the control and treatment groups, their average pro-women attitudes are not statistically distinguishable from zero.

Besides using three separate samples, we also estimate the effects of the information treatments in one fully interacted model. We regress pro-women attitude in the survey experiment on one dummy variable for each information treatment, respondents’ pro-women attitude in the base scenarios, and four interaction terms of the information treatment times respondents’ pro-women attitude in the base scenarios (e.g., same-effort-X-pro-women-base). This model allows the effect of the information treatment to depend on respondents’ initial pro-women attitude in a more fine-grained way. While with using three separate samples we allowed for the effect of the information treatment to differ between each group of respondents, a model with interaction terms allows for the effect to be larger within each group as well. If respondents engage in statistical fairness discrimination, we would expect the coefficients of the interaction terms to be negative to capture that the effects of the information treatments are more negative for respondents who show a larger pro-women attitude in the base scenarios.

Column (1) of Supplementary Appendix Table 2 shows that none of the main effects of the information treatments nor any of the interaction terms are statistically significant. Furthermore, the *F*-test for joint significance does not reject the null hypothesis that all four included interaction terms are equal to zero (*p*-value: 0.1360). Also with this way of estimating the effect of the information treatments, we see no evidence for statistical fairness discrimination.

#### Beliefs About Discriminated Women and Men

**FIGURE 7:**
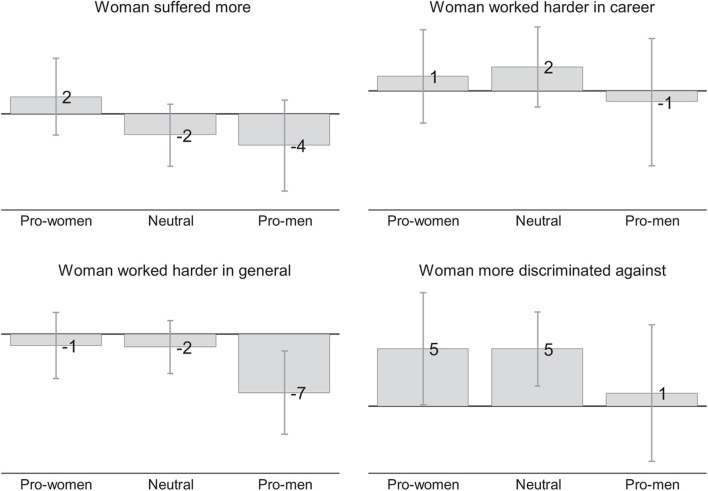
Beliefs about women and men in control scenarios (Qualtrics). This figure is based on responses of Qualtrics respondents who were randomly assigned to the control group. The numbers shown in the figure show belief scores which can range from –50 to +50 where 0 indicates gender neutrality and positive values mean that the statement in the subfigure heading applies more to women. For example, positive values in the left-most figure mean that respondents believe that the women showed in the previous scenarios in the survey experiment would suffer more from not getting the job while negative values mean the opposite. Vertical lines show 95 percent confidence intervals.

The gray bars in Figure 7 show the beliefs of the control group’s respondents about the women and men in the scenarios separately for pro-women, neutral, and pro-men respondents. The numbers reported in the figure are based on scales that range from –50 points to +50 points, where 0 indicates gender neutrality and higher values that the statements shown in the figure apply more to women.

The beliefs about gender differences are weak and often not statistically significant. However, the direction of the point estimates is broadly consistent with our predictions: Pro-women respondents believe that the women in the scenarios would suffer more from not getting the job, worked harder in their career (but not in general), and would have suffered more discrimination. Pro-men respondents believe that men would suffer more from not getting the job, and worked harder in their career and in general. However, they also believe that women suffered more discrimination.

#### Replication With Mturk Sample

**FIGURE 8:**
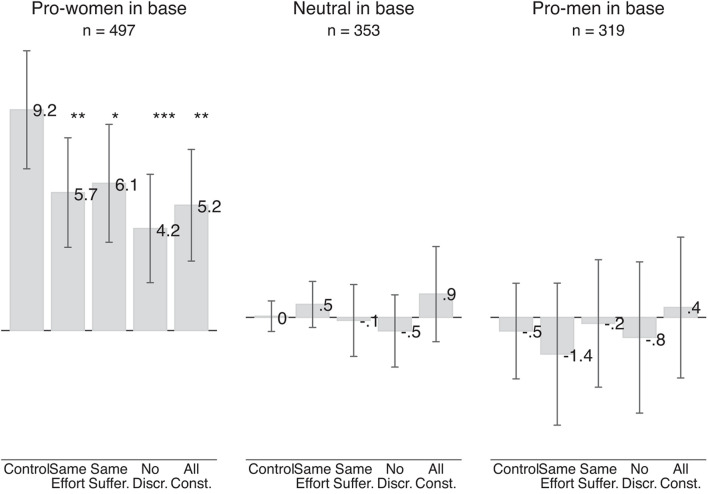
Effect of information treatments on pro-women attitude (Mturk sample). The gray bars show the average pro-women attitude in a given treatment. Each bar in the control group shows the average pro-women attitude for pro-women, neutral and pro-men respondents, respectively. The left-most bar is based on 2,144 judgments made by 268 respondents who were pro-women in the base scenarios and were randomly assigned to the control group, judging four pairs of scenarios involving two judgments each. Each of the next four bars is based on 458 judgments made by 229 respondents who were pro-women in the base scenarios and were randomly assigned to any of the three treatment groups. Similarly, the left-most bar in the middle section is based on 340 judgments by 170 respondents who were neutral in the base scenario, and each of the next four bars is based on 366 judgments made by 183 respondents. The left-most bar in the right-most section is based on 340 judgments made by 170 respondents who were pro-men in the base scenario, and the next four are based on 298 judgments by 149 respondents. Vertical lines show 95 percent confidence intervals based on standard errors clustered at the individual level. Statistical significance of the difference between control group and scenarios with different information treatments is denoted by ***p-value < 0.01, **p-value < 0.05, and *p-value < 0.10.

While we did not elicit their beliefs, we did run the survey experiment with Mturk respondents. Figure 8 shows the results of this survey experiment.

In contrast to our results with the Qualtrics sample, we do see significant treatment effects for pro-women respondents. In the control scenarios, the average pro-women attitude of respondents who we classified as pro-women based on their answer to the base scenarios is 9.2 points. In the treatment scenarios, the pro-women attitude is between 3.1 points and 5.0 points lower. These differences are statistically significant at the 5 percent level for the “same effort” scenarios, “all constant” scenarios, and significant at the 1 percent level for the “no discrimination” scenarios.

Our results for neutral and pro-men respondents are similar to the results in the Qualtrics sample. We see no significant effect of the information treatment for either group of respondents. The absence of the treatment effect for pro-men respondents is again driven by an increase in the pro-women attitude (i.e., a reduction in pro-men attitude) in the control group.

When we estimate the effect of the information treatments using one fully interacted model we see that the effect of the “all constant” treatment is significantly more negative for respondents who showed a larger pro-women attitude in the base scenario (see Column 2 of Supplementary Appendix Table 2). Also with this empirical approach we find some evidence that Mturk respondents engage in statistical fairness discrimination.

### Summary of Results and Discussion

The results of the survey experiment only present mixed evidence for the statistical fairness discrimination explanation. In our main sample, we see that holding constant additional information on characteristics that may explain gender differences in deservingness does not significantly affect respondents’ pro-women attitude. In our replication sample we find effects that are statistically significant and go in the expected direction for pro-women respondents but not for pro-men respondents.

The difference in the effect of the information treatment between the two samples might be driven by differences in underlying beliefs. In the Qualtrics sample, respondents’ beliefs showed that gender of the victim may not have been a useful signal for inferring applicants’ deservingness. While pro-women respondents in this sample believe that the woman (compared to the man) described in the scenarios would suffer more from not getting the job, worked harder in their career, and would have suffered more discrimination, the magnitude of these differences is small. It is therefore not surprising that explicitly holding those factors constant did not have much of an effect on respondents’ pro-women attitude. In the Mturk sample, the meaningful treatment effects might have been driven by gender being a stronger signal for candidate’s deservingness. For example, the significant effect of the “same effort” treatment might be driven by pro-women respondents in the Mturk sample believing that the women described in the scenarios worked much harder in their career.

Differences in beliefs between samples and contexts could also explain why our results differ from those reported by Cappelen et al. (2019). In their context, subjects may believe that men who lose have simply not worked hard enough and are therefore less deserving of benefiting from redistribution. The experimental manipulation of determining winners and losers by chance may have affected people’s decision by ruling out this reason for treating men and women differently.

While we believe that statistical fairness discrimination matters, using gender to draw inferences about deservingness is unlikely to be the only reason for differences in judgments about discriminated women and men. In both of our samples, we still see significant levels of pro-women attitude in scenarios for which we have explicitly held suffering, effort *and* discrimination constant (Qualtrics sample: 6.1 point, Mturk sample 5.2 points). While we cannot rule out that the remaining pro-women attitude is completely driven by beliefs about other unobserved characteristics of the victim, we do not think this is plausible. Instead, we find it more likely that respondents judge discriminators according to factors other than the victim’s deservingness, such as the inferred intentions of the manager. For example, respondents may have assumed that a manager who discriminates against women may have bad intentions (e.g., sexism) whereas a manager who discriminates against men may have good intentions (e.g., increasing gender equality).

## Conclusion

We have shown that even in apparently identical scenarios people judge discrimination against women less harshly than discrimination against men. We have further investigated to what extent this gender gap is driven by what Cappelen et al. (2019) have termed “statistical fairness discrimination.” Our results only lend mixed support for this mechanism: the use of gender as a signal for the victim of discrimination’s deservingness is unlikely to account for the whole pro-women attitude. However, the victim of the gender may have been used as a signal for other relevant characteristics such as the intention of the discriminator.

All our results and conclusions are based on variations of the same generic scenario. We hope that future research establishes to what extent people show a pro-women attitude in other scenarios as well. Some factors that might affect the pro-women attitude are the gender of the manager (which we did not specify), whether the job is in a predominantly male or female industry, and the social status of the job.

The concept of statistical fairness discrimination also inspires promising avenues for future research. Could this practice, for example, explain why people give less harsh sentences for women than men who committed similar crimes (Shatz and Shatz, 2012)? If it does, what are the differences in beliefs that are driving this? To answer these and related questions, researchers could follow our approach of measuring beliefs about unobserved gender differences and randomly holding additional information constant.

## Data Availability Statement

The data and Stata do-files to replicate our results are made available at osf.io/2eq43.

## Ethics Statement

The studies involving human participants were reviewed and approved by Victoria University of Wellington Human Ethics Committee. Written informed consent for participation was not required for this study in accordance with the national legislation and the institutional requirements.

## Author Contributions

EF, JF, and SN designed the experiment and wrote the manuscript. SN programmed the survey, collected and cleaned the data. JF did the analysis. All the authors contributed to the article and approved the submitted version.

## Conflict of Interest

The authors declare that the research was conducted in the absence of any commercial or financial relationships that could be construed as a potential conflict of interest.

## Publisher’s Note

All claims expressed in this article are solely those of the authors and do not necessarily represent those of their affiliated organizations, or those of the publisher, the editors and the reviewers. Any product that may be evaluated in this article, or claim that may be made by its manufacturer, is not guaranteed or endorsed by the publisher.

## Footnotes

1 We pre-registered our study at socialscienceregistry.org (Feess et al., 2021).

2 The age bins and sampling targets were: 18–24 years (12.4%), 25–34 years (17.9%), 35–44 years (16.35%), 45–54 years (17.1%), 60–64 years (7.9%), 65+ years (19.8%).

3 Regression to the mean can also explain the decrease of pro-women attitude of the pro-women respondents from 22.4 points in the base scenario to 6 points in the survey experiment.
