# Supplementary material for: People Judge Discrimination Against Women More Harshly Than Discrimination Against Men – Does Statistical Fairness Discrimination Explain Why?
Source: Front Psychol. 2021 Sep 20;12:675776. doi: 10.3389/fpsyg.2021.675776 (PMC8488152; doi:10.3389/fpsyg.2021.675776)
Supplement: Supplementary file 1 [file Data_Sheet_1.pdf]

Appendix B: Questionnaire

The figure below shows the structure of the questionnaire.

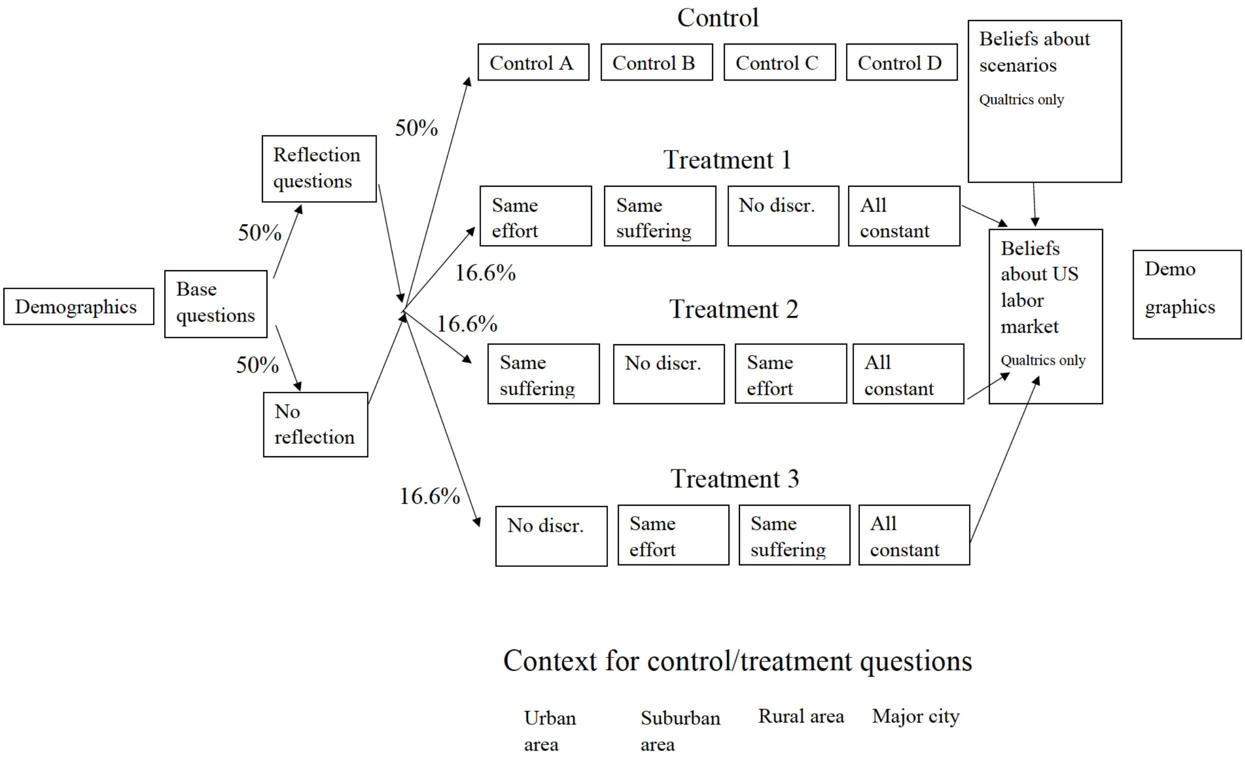

From the next page onwards, we show the questionnaire text.

This is an anonymous survey; we do not record identifiable information.

What is your sex?

- ◯ Male (1)
- ◯ Female (2)

Do you live in the United States of America?

- ◯ Yes (1)
- ◯ No (2)

*

How old are you (in years)?

____

What is your highest degree of completed education?

- ◯ Less than high school diploma (1)
- ◯ High school diploma/GED (2)
- ◯ Some college (no degree) (3)
- ◯ Associate's degree (4)
- ◯ Bachelor's degree (5)
- ◯ Graduate degree (6)

What is your race?

- ◯ White (1)
- ◯ Black (2)
- ◯ American Indian or Alaska Native (3)
- ◯ Asian or Pacific Islander (4)
- ◯ Other (8)

What is your religious preference? Is it Protestant, Catholic, Jewish, some other religion, or no religion?

- ◯ Protestant (1)
- ◯ Catholic (2)
- ◯ Jewish (3)
- ◯ No religion (4)
- ◯ Other (5)
- ◯ Don't know (6)

Page Break

Which category best represents your household's total combined income (before tax) during the past 12 months?

- ○ Less than $5,000 (1)
- ○ $5,000 to $14,999 (2)
- ○ $15,000 to $24,999 (3)
- ○ $25,000 to $34,999 (4)
- ○ $35,000 to $49,999 (5)
- ○ $50,000 to $74,999 (6)
- ○ $75,000 to $99,999 (7)
- ○ $100,000 to $149,999 (8)
- ○ $150,000 or more (9)

What best describes your current situation?

- ○ Employed full time (1)
- ○ Employed part time (2)
- ○ Self-employed (3)
- ○ Studying (4)
- ○ Looking for a job (5)
- ○ Retired (7)
- ○ Homemaker (8)
- ○ Other (please specify) (6)

In politics, as of today, do you consider yourself a Republican, a Democrat, or an independent?

- ○ Republican (1)
- ○ Democrat (2)
- ○ Independent (3)
- ○ Other party/don't know (7)

Page Break

End of Introduction

Start of Base Questions

We will now ask you to evaluate several hiring decisions from a moral perspective, on a scale that ranges from **very morally wrong** to **very morally right**.

For example, most people find stealing morally wrong and donating to charity morally right.

Please think carefully about your answers.

Page Break

[The order of the following two questions is randomized]

Consider the following hypothetical scenario.

A job is available in a company. A manager in the company has narrowed down the pool of applicants to the two most qualified people: one man and one woman.

Taking into account all characteristics of the two applicants (qualifications, experience, personality, etc.), the manager knows that **the woman is slightly more qualified** and hiring her would bring slightly higher profits for the company.

After considering everything, **the manager hires the man.**

I think the decision of the manager is:

	Very morally wrong	Very morally right
	0 10 20 30 40 50 60 70 80 90 100	

Page Break

Consider the following hypothetical scenario.

A job is available in a company. A manager in the company has narrowed down the pool of applicants to the two most qualified people: one man and one woman.

Taking into account all characteristics of the two applicants (qualifications, experience, personality, etc.), the manager knows that **the man is slightly more qualified** and hiring him would bring slightly higher profits for the company.

After considering everything, **the manager hires the woman.**

I think the decision of the manager is:

	Very morally wrong — Very morally right<br>0 10 20 30 40 50 60 70 80 90 100
1 (1)	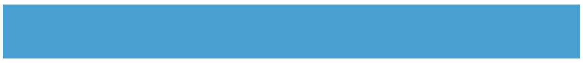

End of Base Questions

Start of Followup Questions (50% of respondents, randomly selected, are shown one of the following questions)

[If the respondent judged discrimination against the woman to be worse in the base scenarios]

We have just shown you two scenarios which were identical in all aspects except for the gender of the person who was more qualified and the gender of the person whom the manager decided to hire.

Your evaluations of the manager's decisions in these two scenarios suggest that:

You find it worse (from a moral perspective) if a manager hires a less qualified man

over a more qualified woman (compared to the other way around).

Is that a correct description of your opinion?

- ◯ Yes, that is correct (1)
- ◯ No, I find both equally bad (or good) (2)
- ◯ No, I find it worse if the manager hires a less qualified woman over a more qualified man (3)

[If the respondent judged discrimination against the man to be worse in the base scenarios]

We have just shown you two scenarios which were identical in all aspects except for the gender of the person who was more qualified and the gender of the person whom the manager decided to hire.

Your evaluations of the manager's decisions in these two scenarios suggest that:

You find it worse (from a moral perspective) if a manager hires a less qualified woman over a more qualified man (compared to the other way around).

Is that a correct description of your opinion?

- ◯ Yes, that is correct (1)
- ◯ No, I find both equally bad (or good) (2)
- ◯ No, I find it worse if the manager hires a less qualified man over a more qualified woman (3)

[If the respondent judged both acts of discrimination in the initial scenario to be equally bad]

We have just shown you two scenarios which were identical in all aspects except for the gender of the person who was more qualified and the gender of the person whom the

manager decided to hire.

Your evaluations of the manager's decisions in these two scenarios suggest that:

You find it equally morally objectionable if a manager hires a less qualified woman over a more qualified man or a less qualified man over a more qualified woman.

Is that a correct description of your opinion?

- ◯ Yes, that is correct (1)
- ◯ No, I find it worse if the manager hires a less qualified woman over a more qualified man (2)
- ◯ No, I find it worse if the manager hires a less qualified man over a more qualified woman (3)

End of Followup Questions

Start of Control Questions

50% of respondents, randomly selected, are shown these questions

Respondents in the control group see four additional versions of the base questions, set in an urban area, suburban area, rural area, and major city, in that order. Each pair of questions is separated by a page break.

We will now ask you to consider several similar scenarios. The differences to the first two scenarios are marked in yellow.

Consider the following hypothetical scenario.

A job is available in a company in a[n] [urban area/suburban area/rural area/major city]. A manager in the company has narrowed down the pool of applicants to the two most qualified people: one man and one woman.

Taking into account all characteristics of the two applicants (qualifications, experience, personality, etc.), the manager knows that **the woman is slightly more qualified** and hiring her would bring slightly higher profits for the company.

After considering everything, **the manager hires the man.**

I think the decision of the manager is:

Very morally wrong										Very morally right
0	10	20	30	40	50	60	70	80	90	100

1 (1)

Consider the following hypothetical scenario.

A job is available in a company in a[n] [urban area/suburban area/rural area/major city]. A manager in the company has narrowed down the pool of applicants to the two most qualified people: one man and one woman.

Taking into account all characteristics of the two applicants (qualifications, experience, personality, etc.), the manager knows that **the man is slightly more qualified** and hiring him would bring slightly higher profits for the company.

After considering everything, **the manager hires the woman.**

I think the decision of the manager is:

	Very morally wrong	Very morally right
	0 10 20 30 40 50	60 70 80 90 100
1 (1)		

Page Break

End of Control Questions

Start of Treatment Questions

50% of respondents, randomly selected, are shown these questions

For the first three treatment questions, the pair [factor being held constant, type of area] is randomised. More specifically:

16.6% of respondents see:

1. Same effort in an urban area
2. Same suffering in a suburban area
3. No discrimination in a rural area
4. All constant in a major city

16.6% see:

1. Same suffering in an urban area

2. No discrimination in a suburban area
3. Same effort in a rural area
4. All constant in a major city

Finally, 16.6% see:

1. No discrimination in an urban area
2. Same effort in a suburban area
3. Same suffering in a rural area
4. All constant in a major city

Consider the following hypothetical scenario.

A job is available in a company in a[n] [urban/suburban/rural] area. A manager in the company has narrowed down the pool of applicants to the two most qualified people: one man and one woman.

Taking into account all characteristics of the two applicants (qualifications, experience, personality, etc.), the manager knows that **the woman is slightly more qualified** and hiring her would bring slightly higher profits for the company.

The manager knows that the man and the woman would suffer equally from not getting the job. For example, both are currently unemployed, but have enough savings so that they could go without getting a paycheck for another four weeks. Also, both would find it equally hard to get a new job. Neither of them has to support a family.

After considering everything, **the manager hires the man.**

I think the decision of the manager is:

Very morally wrong   Very morally right

0 10 20 30 40 50 60 70 80 90 100

1 (1)

Consider the following hypothetical scenario.

A job is available in a company in a[n] [urban/suburban/rural] area. A manager in the

company has narrowed down the pool of applicants to the two most qualified people: one man and one woman.

Taking into account all characteristics of the two applicants (qualifications, experience, personality, etc.), the manager knows that **the man is slightly more qualified** and hiring him would bring slightly higher profits for the company.

The manager knows that the man and the woman would suffer equally from not getting the job. For example, both are currently unemployed, but have enough savings so that they could go without getting a paycheck for another four weeks. Also, both would find it equally hard to get a new job. Neither of them has to support a family.

After considering everything, **the manager hires the woman.**

I think the decision of the manager is:

Very morally wrong Very morally right

0 10 20 30 40 50 60 70 80 90 100

1 (1)

Page Break

Consider the following hypothetical scenario.

A job is available in a company in a[n] [urban/suburban/rural] area. A manager in the company has narrowed down the pool of applicants to the two most qualified people: one man and one woman.

Taking into account all characteristics of the two applicants (qualifications, experience, personality, etc.), the manager knows that **the woman is slightly more qualified** and hiring her would bring slightly higher profits for the company.

The manager knows that the job is in an industry where there is no gender discrimination. A number of studies have convincingly shown that in this industry neither men nor women face discrimination in hiring decisions, nor do they face any other unfair treatment by coworkers or supervisors because of their gender.

After considering everything, **the manager hires the man.**

I think the decision of the manager is:

Very morally wrong Very morally right

0 10 20 30 40 50 60 70 80 90 100

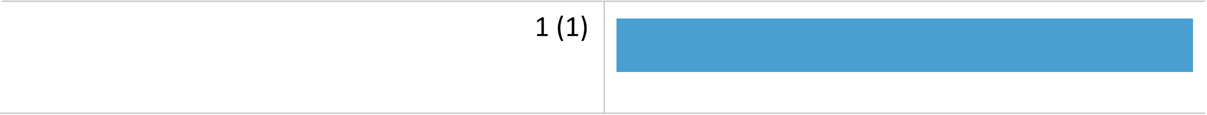

Consider the following hypothetical scenario.

A job is available in a company in a[n] [urban/suburban/rural] area. A manager in the company has narrowed down the pool of applicants to the two most qualified people: one man and one woman.

Taking into account all characteristics of the two applicants (qualifications, experience, personality, etc.), the manager knows that **the man is slightly more qualified** and hiring him would bring slightly higher profits for the company.

The manager knows that the job is in an industry where there is no gender discrimination. A number of studies have convincingly shown that in this industry neither men nor women face discrimination in hiring decisions, nor do they face any other unfair treatment by coworkers or supervisors because of their gender.

After considering everything, **the manager hires the woman.**

I think the decision of the manager is:

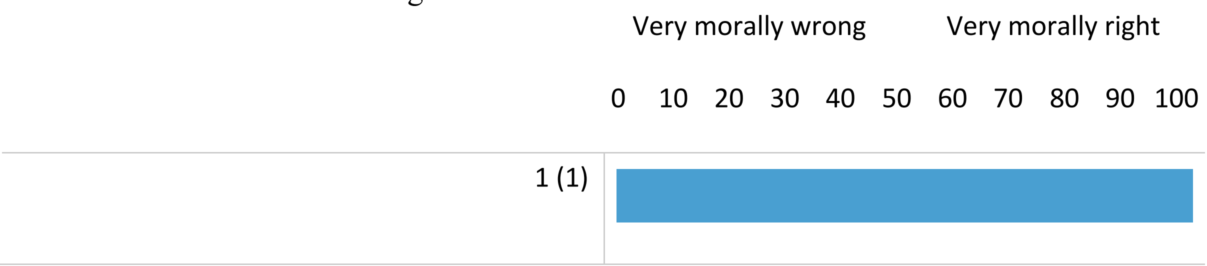

Page Break

Consider the following hypothetical scenario.

A job is available in a company in a[n] [urban/suburban/rural] area. A manager in the company has narrowed down the pool of applicants to the two most qualified people: one man and one woman.

Taking into account all characteristics of the two applicants (qualifications, experience, personality, etc.), the manager knows that **the woman is slightly more qualified** and hiring

her would bring slightly higher profits for the company.

The manager knows that the man and the woman have worked equally hard in their career. For example, both regularly studied on the weekends while their friends were out partying.

After considering everything, **the manager hires the man.**

I think the decision of the manager is:

	Very morally wrong	Very morally right
	0 10 20 30 40 50 60 70 80 90 100	
1 (1)		

Consider the following hypothetical scenario.

A job is available in a company in a[n] [urban/suburban/rural] area. A manager in the company has narrowed down the pool of applicants to the two most qualified people: one man and one woman.

Taking into account all characteristics of the two applicants (qualifications, experience, personality, etc.), the manager knows that **the man is slightly more qualified** and hiring him would bring slightly higher profits for the company.

The manager knows that the man and the woman have worked equally hard in their career. For example, both regularly studied on the weekends while their friends were out partying.

After considering everything, **the manager hires the woman.**

I think the decision of the manager is:

	Very morally wrong	Very morally right
	0 10 20 30 40 50 60 70 80 90 100	
1 (1)		

Page Break

Consider the following hypothetical scenario.
A job is available in a company in a major city. A manager in the company has narrowed down the pool of applicants to the two most qualified people: one man and one woman.

Taking into account all characteristics of the two applicants (qualifications, experience, personality, etc.), the manager knows that **the woman is slightly more qualified** and hiring her would bring slightly higher profits for the company.

The manager knows that

- the man and the woman **have worked equally hard in their career,**
- the man and the women **would suffer equally from not getting the job,** and
- the job is in an industry **with no gender discrimination.**

After considering everything, **the manager hires the man.** I think the decision of the manager is:

Very morally wrong Very morally right

0 10 20 30 40 50 60 70 80 90 100

1 (1)

Consider the following hypothetical scenario.
A job is available in a company in a major city. A manager in the company has narrowed down the pool of applicants to the two most qualified people: one man and one woman.

Taking into account all characteristics of the two applicants (qualifications, experience, personality, etc.), the manager knows that **the man is slightly more qualified** and hiring him would bring slightly higher profits for the company.

The manager knows that

- the man and the woman **have worked equally hard in their career,**
- the man and the women **would suffer equally from not getting the job,** and
- the job is in an industry **with no gender discrimination.**

After considering everything, **the manager hires the woman.**

I think the decision of the manager is:

Very morally wrong Very morally right

0 10 20 30 40 50 60 70 80 90 100

1 (1)

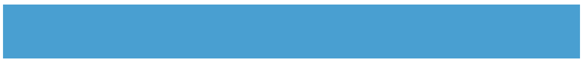

Page Break

End of Treatment Questions

The following four questions were only shown to respondents of the control group in the Qualtrics sample.

We will now ask you a few questions about your beliefs about situations like the ones described in the previous eight scenarios.

In the previous scenarios, do you think the women or the men would **suffer more from not getting the job?**

The women would suffer much more	They would suffer equally	The men would suffer much more

0 10 20 30 40 50 60 70 80 90 100

4 (4)

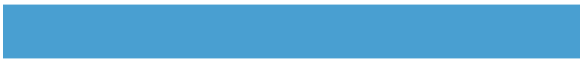

In the previous scenarios, do you think the women or the men **worked harder to get where they are in their career?**

The women worked much harder	They worked equally hard	The men worked much harder

0 10 20 30 40 50 60 70 80 90 100

4 (4)

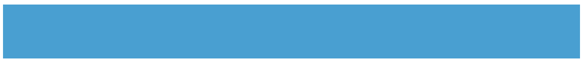

In the previous scenarios, do you think the women or the men are **generally more hard-working (in their career and other aspects of their life)?**

	The women are much more hard-working	They are equally hard-working	The men are much more hard-working

0 10 20 30 40 50 60 70 80 90 100

4 (4)

In the previous scenarios, do you think the women or the men are **more discriminated against in the labor market?**

	The women are much more discriminated against	They are equally discriminated against	The men are much more discriminated against

0 10 20 30 40 50 60 70 80 90 100

4 (4)

The following four questions were only shown to respondents in the Qualtrics sample.

We will now ask you a few questions about the US labour market.

In general, do you think women or men suffer more when they fail to get a job they applied for?

	Women suffer much more	They suffer equally	Men suffer much more

0 10 20 30 40 50 60 70 80 90 100

4 (4)

In general, do you think women or men have to work harder to reach the same career position?

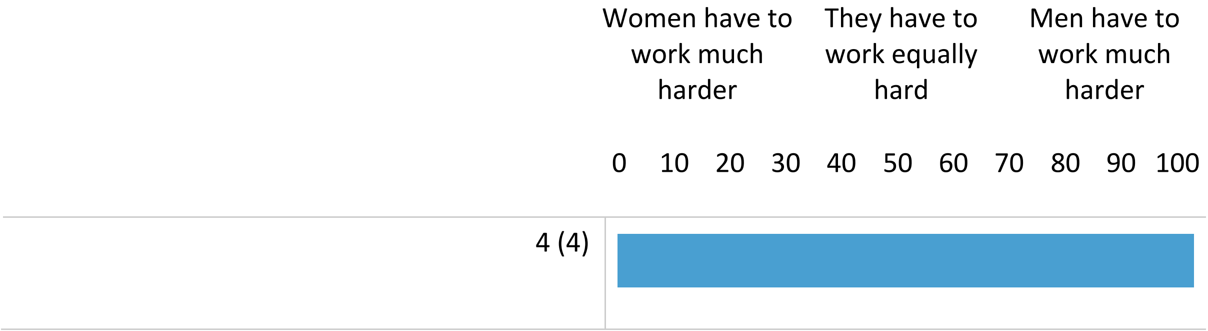

In general, do you think women or men are more hard-working (in their careers and in all aspects of their life)?

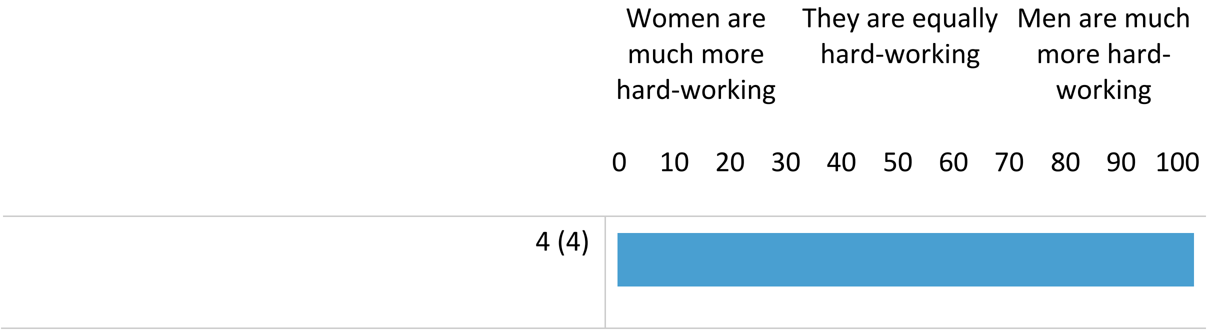

In general, do you think women or men are more discriminated against in the labor market?

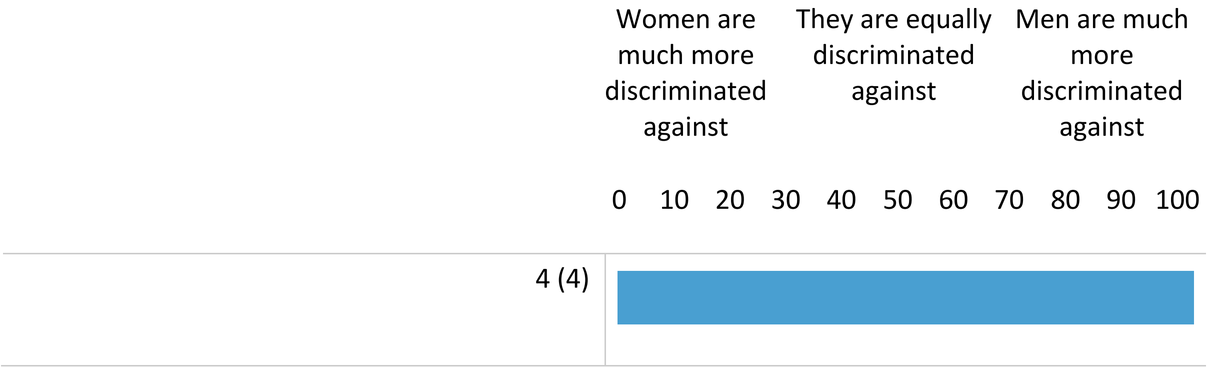

Start of Final Questions (shown to Qualtrics and Mturk sample)

Everything else being equal, do you care more about the wellbeing of men or women?

- ◯ I care more about the wellbeing of men (1)
- ◯ I care more about the wellbeing of women (2)
- ◯ I care equally about the wellbeing of men and women (3)

Are you a feminist?

- ◯ Yes (1)
- ◯ No (2)

Is there anything else you would like to tell us?

____

End of Survey
