# Supplementary material for: People Judge Discrimination Against Women More Harshly Than Discrimination Against Men – Does Statistical Fairness Discrimination Explain Why?
Source: Front Psychol. 2021 Sep 20;12:675776. doi: 10.3389/fpsyg.2021.675776 (PMC8488152; doi:10.3389/fpsyg.2021.675776)
Supplement: Supplementary file 2 [file Table_1.pdf]

Table A1: Summary statistics Mturk Sample (N=1,169)

	(1)	(2)	(3)	(4)
	mean	sd	min	max
Age	37.12	11.87	18	78
Female	0.50	0.50	0	1
Race				
White	0.77	0.42	0	1
Black	0.15	0.36	0	1
Asian or Pacific Islander	0.05	0.22	0	1
Amer. Indian or Alaska Native	0.01	0.12	0	1
Other	0.02	0.12	0	1
Educational attainment				
High school or less	0.19	0.40	0	1
Some college	0.19	0.39	0	1
Associates degree	0.09	0.29	0	1
Bachelor's degree	0.35	0.48	0	1
Graduate degree	0.18	0.38	0	1
Political orientation				
Democrat	0.40	0.49	0	1
Republican	0.39	0.49	0	1
Independent	0.19	0.39	0	1
Other	0.02	0.15	0	1

Note: These summary statistics are based on our Mturk sample.
