# Supplementary material for: People Judge Discrimination Against Women More Harshly Than Discrimination Against Men – Does Statistical Fairness Discrimination Explain Why?
Source: Front Psychol. 2021 Sep 20;12:675776. doi: 10.3389/fpsyg.2021.675776 (PMC8488152; doi:10.3389/fpsyg.2021.675776)
Supplement: Supplementary file 3 [file Table_2.pdf]

## Table A2: Effect of information treatment in fully interacted model

| | (1)<br>Pro-women-attitude | (2)<br>Pro-women-attitude |
|---|---|---|
| Same effort | -1.62 | -0.60 |
| | (1.285) | (0.728) |
| Same suffering | -0.66 | -0.34 |
| | (1.481) | (0.749) |
| No discrimination | -1.97 | -1.14 |
| | (1.488) | (0.773) |
| All constant | 0.60 | 0.14 |
| | (1.292) | (0.813) |
| Pro-Women-base | 0.21* | 0.49*** |
| | (0.118) | (0.063) |
| Same effort-X-pro-women-base | 0.04 | -0.14 |
| | (0.151) | (0.100) |
| Same suffering-X-pro-women-base | -0.05 | -0.13 |
| | (0.153) | (0.101) |
| No discrimination-X-pro-women-base | 0.12 | -0.21* |
| | (0.154) | (0.106) |
| All constant-X-pro-women-base | -0.14 | -0.25** |
| | (0.137) | (0.108) |
| Constant | 1.90** | 1.55*** |
| | (0.886) | (0.442) |
| Observations | 1,912 | 4,676 |
| R-squared | 0.066 | 0.210 |
| F-test interactions, p-value: | 0.136 | .1365 |
| Sample | Qualtrics | Mturk |

**Note:** The dependent variable in both columns in the within-subject pro-women attitude based on respondents' judgments in the survey experiment. Pro-women attitude is the moral judgment of a manger who discriminates against a woman (on a -50 to +50 scale where higher values indicate more disapproval) minus the moral judgment of a manager who discriminates against a man. Positive values mean that respondents evaluate discrimination against a woman as more morally bad. Column (1) is estimated with the Qualtrics sample and Column (2) is estimated with the Mturk sample. The second to last row shows p-values of an F-test for joint significance of all four interaction terms. Standard errors are clustered at the individual level. Statistical significance is denoted by *** p-value<0.01, ** p-value<0.05, and * p-value<0.10.
